# Effect of carbon monoxide on *Mycobacterium tuberculosis* pathogenesis

**DOI:** 10.1186/2045-9912-2-30

**Published:** 2012-12-17

**Authors:** Vineetha M Zacharia, Michael U Shiloh

**Affiliations:** 1 Department of Medicine, Division of Infectious Diseases, University of Texas Southwestern Medical Center, Dallas, TX, 75229-9113, USA; 2 Department of Microbiology, Division of Infectious Diseases, University of Texas Southwestern Medical Center, Dallas, TX, 75229-9113, USA

**Keywords:** Carbon monoxide, Heme oxygenase, Microbiology, Immunology, Mycobacterium tuberculosis, Microbial pathogenesis

## Abstract

The intracellular pathogen *Mycobacterium tuberculosis* (Mtb) is exposed to multiple host antimicrobial pathways, including toxic gases such as superoxide, nitric oxide and carbon monoxide (CO). To survive, mycobacteria evolved mechanisms to resist the toxic environment, and in this review we focus on a relatively new field, namely, the role of macrophage heme oxygenase and its enzymatic product CO in Mtb pathogenesis. In particular, we focus on (i) the induction of heme oxygenase during Mtb infection and its relevance to Mtb pathogenesis, (ii) the ability of mycobacteria to catabolize CO, (iii) the transcriptional reprogramming of Mtb by exposure to CO, (iv) the general antimicrobial properties of CO and (v) new genetic evidence characterizing the ability of Mtb to resist CO toxicity. Developing a complete molecular and genetic understanding of the pathogenesis of Mtb is essential to its eventual eradication.

## Introduction

The success of a pathogen during infection depends upon its abilities to respond to and overcome a battery of host defense mechanisms. In response to bacterial infection, host cells generate a variety of toxic compounds to mediate microbial killing such as excess hydrogen ion (H+), hydrogen peroxide (H_2_O_2_), hypochlorous acid (HOCl), nitric oxide (NO), and carbon monoxide (CO). To promote intracellular survival, some pathogens such as *Mycobacterium tuberculosis* (Mtb) evolved multiple pathways to evade these host defenses. For example, mycobacteria utilize superoxide dismutase [1] and catalase [2,3] to convert the toxic reactive oxygen intermediates superoxide and H_2_O_2_ to water and oxygen, while they also employ multiple mechanisms to resist nitric oxide toxicity [4-8].

**Figure 1:**
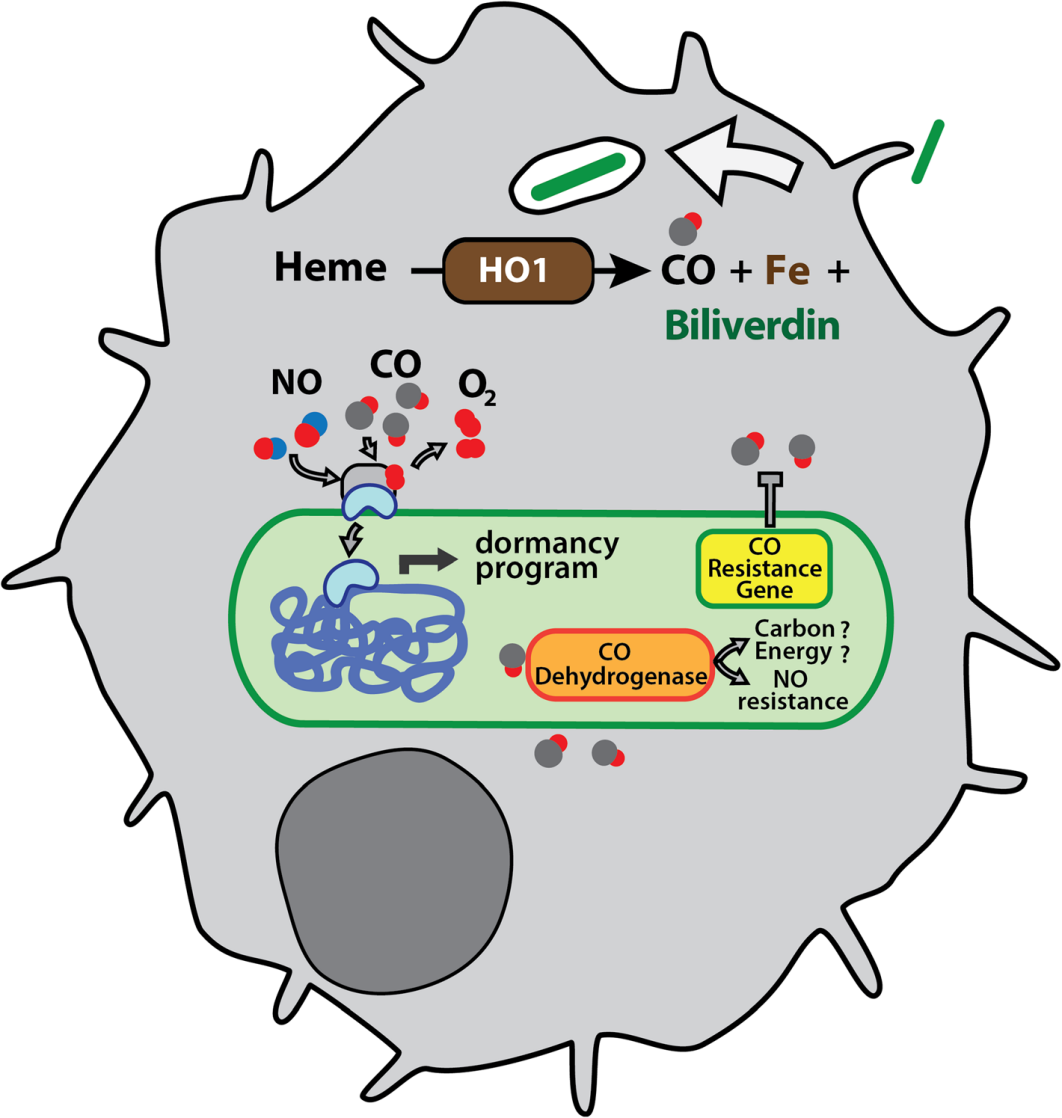
**Role of carbon monoxide in M. tuberculosis pathogenesis.** Macrophage infection by Mtb induces HO-1. HO-1 catabolizes heme to release CO, iron and bilverdin. CO produced by HO1 can alter Mtb gene transcription by activating the DosS/DosR two component signal transduction system to stimulate a dormancy program. CO-mediated growth inhibition is resisted by the expression of a genetically encoded Mtb gene. Some mycobacteria can catabolize CO via CO dehydrogenase for growth. Alternatively, CODH may function in resisting host-derived nitric oxide.

Understanding Mtb resistance mechanisms against host defenses is of paramount importance as it is an endemic and epidemic pathogen that latently infects approximately one-third of the world’s population [9]. Upon Mtb infection, host immune pathways are activated, resulting in macrophage and T cell recruitment [10]. The long-term success of Mtb as an intracellular pathogen lies primarily in its ability to remain dormant and persist within host macrophages for extended periods of time. This is facilitated in part by the induction of genes that comprise the dormancy regulon by stimuli present in the Mtb microenvironment including low oxygen, NO, nutrient starvation, and CO (Figure 1) [11-14]. The genes in the dormancy regulon, many which are of unknown function, likely contribute to TB persistence by facilitating its long-term survival [15].

Recent studies have described the deleterious effects of CO on various microbes, while unveiling the potential bacterial targets of CO action. In *Escherichia coli*, *Pseudomonas aeruginosa*, and *Staphylococcus aureus*, exposure to CO inhibits key enzymes of the electron transport chain required for bacterial respiration, resulting in microbial death [16,17]. In contrast to the aforementioned organisms, Mtb is able to withstand high concentrations of CO, suggesting a potential CO resistance pathway not previously described in microorganisms [11]. In this review, we describe the role of the reactive gas compound CO and its relevance during microbial infection, while highlighting the ability of Mtb to withstand CO toxicity.

### Mtb infection increases heme oxygenase expression

In humans and mice, three isoforms of heme oxygenase exist, HO-1, HO-2, HO-3 (encoded by *Hmox1*, *Hmox2*, and *Hmox3* genes, respectively). All three isoforms catabolize heme, releasing as products free iron, biliverdin and CO. HO-2 and HO-3 are constitutively expressed, whereas HO-1 is induced by bacterial lipopolysaccharide, hypoxia, tumor necrosis factor (TNF), reactive nitrogen and oxygen intermediates [18,19] and also by Mtb infection [11,20]. Upregulation of HO-1 may benefit host cells since CO and biliverdin/bilirubin can act as signaling molecules as well as provide cytoprotection. CO contributes to the cytoprotective effects of HO-1 by preventing free heme accumulation within cells, suppressing endothelial cell apoptosis, and modulating an anti-inflammatory response in macrophages upon exposure to bacterial lipopolysaccharide [21-23]. Likewise, both biliverdin and bilirubin (under the influence of biliverdin reductase) can protect cells from a variety of cytotoxic insults [24].

Notably, HO-1 deficient mice manifest decreased ability to overcome pathogenic infection and to recover from inflammatory diseases, xenotransplantation, and heart diseases (reviewed in [25]). In humans, a polymorphism in the *Hmox1* promoter result in differential expression of HO-1 such that individuals with fewer (GT)n repeats in the *Hmox1* promoter transcribe more HO-1 in response to various stimuli, resulting in enhanced protection from both infectious and non-infectious diseases [26]. This strongly indicates that robust cellular HO-1 expression is crucial to overcome infectious and non-infectious diseases by mediating a wide range of host regulatory pathways.

Previously, we [11] and others [20] found that during Mtb infection, HO-1 is induced in both infected macrophages and mice suggesting that increased levels of CO might be present during Mtb infection (Figure 1) [11]. This induction occurred very early during mouse infection, i.e. within 10 days, and was concentrated in nascent granuloma and tissue macrophages [11]. The precise signaling mechanism of HO-1 induction by Mtb is unknown, though bacterial factors, free heme, and inflammatory cytokines likely combine to induce *HO-1* transcription. Although the exact concentration of CO in lungs during Mtb infection is not known, CO concentrations can range from 2–50 ppm, depending on the physiologic status of the individual. Thus, the average, nonsmoking human exhales approximately 2 ppm [27,28] while patients with a variety of infectious and inflammatory conditions producing significantly more [27,29-32].

What might be the function of HO-1 during infection? Considering that the induction is robust at the direct site of infection, i.e. macrophages within granuloma, it is feasible that HO-1 may be involved in controlling Mtb growth. Given the pleiotropic signaling activity of HO-1 and CO, other mechanisms might also be HO-1/CO dependent during Mtb infection. For example, HO-1 enhances interferon regulatory factor 3 (IRF3) phosphorylation and interferon-β (IFN-β) production in Listeria or virally infected macrophages [33] and Mtb infection of macrophages rapidly induces IRF3 phosphorylation and IFN-β production [34]. Thus, the observed activation of the IRF-3/IFN-β pathway during Mtb infection [34] may also be HO-1 dependent. In addition to regulating cytokine production, HO-1 and CO may also be involved in triggering the autophagy pathway for eradication of intracellular bacteria termed xenophagy [35]. Autophagy plays a major role in controlling Mtb infection infection [36,37] and recent work found that inhibition of HO-1 prevented endotoxin-induced autophagy [38], suggesting that during Mtb infection, upregulation of HO-1 with concomitant CO production enhances multiple innate immunity mechanisms**.**

### Carbon monoxide as a carbon and electron source in mycobacteria

Albeit a toxic gas, carbon monoxide also functions as an intermediate molecule in bacterial metabolic pathways. Certain aerobic and anaerobic microorganisms, particularly those that utilize CO as the sole carbon and energy source (carboxydotrophs), employ the enzyme carbon monoxide dehydrogenase (CODH) to convert reactive carbon monoxide into more stable compounds [39]. Specifically, CODH catalyzes the reaction CO + H_2_O -> CO_2_ + 2e^-^ + 2H^+^ when organic carbon is absent (autotrophic growth) and carbon monoxide is present [40]. CO dehydrogenase is a complex metalloprotein composed of 3 polypeptides. In the carboxydotroph *Oligotropha carboxydovorans*, the three structural genes of CODH are *coxL* (for CO oxidation protein, Large subunit), *coxM* (medium subunit) and *coxS* (small subunit) (Figure 1). The entire *cox* cluster is transcriptionally induced when the bacteria are grown under autotrophic conditions in the presence of CO but not under heterotrophic conditions (organic carbon rich) [40]. Although the mechanism of this transcriptional induction remains unknown, these genes are necessary for autotrophic growth [41]. In aerobes, CODH coordinates molybdenum in its active site to oxidize CO to CO_2_ and the electrons generated from the oxidation reaction is transferred to the final electron acceptor such as ferredoxin, cytochromes, FMN or FADH_2_, which are then subsequently coupled to other energy requiring processes [42,43]. CODH in anaerobic microbes also catalyzes CO oxidation, but instead of coordinating molybdenum in its active site, it contains a Ni-Fe active site. When coupled to acetyl-CoA synthase (ACS), CODH converts CO_2_ to CO in the Wood-Ljungdahl pathway for subsequent synthesis of a major carbon source, acetyl-CoA [44,45]. Thus, oxidation of CO can simultaneously produce energy for the cell and additional sources of carbon.

More recent evidence suggests that CO utilization via CODH is widespread among diverse microbial species, including the mycobacterial species *M*. *bovis* BCG, *M. gordonae*, *M*. *smegmatis*, and *M. tuberculosis*[43,46,47]. Mtb encodes for orthologues of CODH subunits [40]. The CODH structural genes are arranged in the transcriptional order 5’ *coxM* (Rv0375c) ->*coxS* (Rv0374c) ->*coxL* (Rv0373c) 3’, a genome structure shared by the majority of bacteria with *cox* homologues [40]. All three of the putative Mtb CODH proteins demonstrate high overall sequence similarity with *O. carboxydovorans* and all sequenced mycobacterial genomes including that of *M. avium*, *M. bovis*, *M. leprae*, and *M. smegmatis* encode for *cox* homologues with extremely high sequence similarity to Mtb [48]. Notably, as more genomes have been sequenced, *cox* homologues have been identified in several additional pulmonary pathogens, including *Burkholderia sp*., *Rhodococcus sp*., and *Pseduomonas sp*. (our unpublished observations).

The identification of *cox* homologues in various mycobacteria species prompted Park et al. to test the ability of mycobacteria to grow *in vitro* on CO as the sole carbon source [48]. Strikingly, all of the mycobacteria tested were able to grow on CO at 30% atmosphere as the sole carbon source, albeit more slowly [48]. Growth on CO required a long lag period after the bacteria were first subjected to CO-growth media, suggesting transcriptional induction of CO utilization genes [48]. Notably, CO-dependent growth of virulent Mtb was not tested. Additionally, Mtb and some of its relatives were found to utilize CO at <1-5 parts per million (ppm), an environmentally and physiologically relevant range since CO in the atmosphere and lungs measure at approximately 0.1 to 0.5 ppm and <3 ppm, respectively [27,47]. To date, no mutants in the *cox* genes have been reported in Mtb. However, that Mtb has retained these large genes during its evolution as a pathogen without a known *ex vivo* existence suggest that Mtb might utilize CO as an alternative carbon source, which may confer a selective advantage for Mtb within the nutrient-limited confines of a macrophage. An alternative explanation may be that the *cox* genes serve another function, namely, nitric oxide detoxification [49]. Although recombinant CODH from mycobacteria was able to oxidize NO and protect *E. coli* from NO mediated toxicity [49], direct genetic evidence that the *cox* genes are required by Mtb in vitro or in vivo to protect Mtb is lacking. Thus, mycobacterial CODH may have at least two activities, namely, CO uptake and NO detoxification, and further pathogenesis assays will be needed to dissect the precise function(s) of Mtb CODH (Figure 1).

### Gene expression of Mtb in the presence of carbon monoxide

Since Mtb resides within the lung, and since CO is exhaled continuously, it is reasonable to predict that Mtb might have evolved mechanisms to detect and respond to changing CO fluxes, partly to sense the host immune status. In fact, both prokaryotes and eukaryotes have developed systems for sensing carbon monoxide [29,50-52]. For example, in eukaryotes the transcription factor NPAS2, implicated in regulating circadian rhythm, was shown to bind CO resulting in decreased DNA binding activity [53]. Likewise, the bacterium *Rhodospirillum rubrum* expresses a CO-binding transcription factor, CooA, whose function is to stimulate production of a CO oxidation system distinct from the one found in *O. carboxydovorans*[54-57]. How do organisms sense and measure CO? Commonly, these proteins contain an associated heme moiety which is not surprising given the propensity of CO to bind heme [50]. However, the physiologic conditions and precise mechanisms used by these proteins to bind both heme and CO are diverse. For instance, CooA from *R. rubrum* can only bind CO when its heme is in the ferrous (Fe^2+^) state, a reduced condition found stably only under purely anaerobic conditions [57-59]. Thus, an organism like Mtb, which expresses a CO oxidation system under aerobic conditions [48] would be unlikely to express a CooA homologue, and in fact no CooA homologue can be identified in the Mtb genome.

To test the response of Mtb to CO, we exposed Mtb to CO in vitro and assessed the effects using transcriptional profiling [11]. We found that CO induces the transcription of a cohort of genes known as the dormancy (dos) regulon [11]. This induction occurred at CO concentrations as low as 20 ppm headspace CO, but was most robust at concentrations above 2000 ppm [11]. Mtb lacking the DosS/DosT two component system was unresponsive to CO, indicating that DosS is the primary sensor for CO. Notably, DosS also sense NO and hypoxia via its heme binding domain (Figure 1) [60]. To confirm CO sensing can occur in vivo, we infected wild-type mouse macrophages and macrophages deficient in *HO-1* and found a significant abrogation of dormancy gene induction in the absence of *HO-1*[11]. Similar results were obtained by Kumar et. al, confirming that Mtb can sense CO in vitro and in vivo [20].

### General antimicrobial properties of carbon monoxide

It has been nearly four decades since preliminary studies have described the antibacterial effects of carbon monoxide. Specifically, CO was found to inhibit DNA replication in *E. coli* and it was postulated that CO may disrupt unwinding of the DNA duplex during replication, rather than directly inhibiting DNA polymerase activity [61]. However, it was later discovered that CO halts DNA replication by reducing the intracellular concentration of ATP and dNTPs. By disrupting enzymes in the electron transport and ATP production pathways, it was found that the presence of CO led to the depletion of deoxynucleoside triphospate pools in *E. coli*[62]. CO was also found to inhibit growth of the airborne bacteria *Serratia marcescens* by causing a flux in energy-generating pathways, namely the electron transport system [63].

Recently there has been revived interest in examining the role of exogenous CO on bacterial growth using lipid-soluble carbon monoxide-releasing molecules (CORMs). The original CORMs were metal carbonyl compounds that release CO at physiologically relevant concentrations in biological systems [64]. More recently, newer CORMs have been synthesized that represent unique chemistry [65] and multiple CORM compounds are effective antimicrobial molecules against both gram negative and gram-positive bacteria. In a recent study by Nobre *et al*., cultures of *E. coli* and *S. aureus* were treated with CORM-2 and CORM-3 under aerobic and anaerobic conditions to determine cell viability [66]. In the presence of either CORM, the strains suffered the toxic effects of CO as marked by a significant reduction of CFU/mL compared to cells not treated with a CORM. Furthermore, the study reveals that the bactericidal effects of CO were observed under both aerobic and anaerobic conditions, indicating that there are additional bacterial targets for CO aside from the components involved in aerobic respiration [66]. The potency of CORMs as antimicrobial compounds is further underscored by a study that described reduced cell viability of laboratory and antibiotic-resistant strains of *P. aeruginosa* when treated with CORM3 [16]. ALF-62, a different class of CO-RM containing molybdenum, and CORM2 were recently tested on *E. coli* to elucidate the mechanism by which CO inhibited bacterial growth [65]. In their study, Tavares *et al.* report an accumulation of endogenous reactive oxygen species (ROS) in the presence of these CORMs and observe rescued growth of CORM treated *E. coli* when supplemented with various antioxidants [65].

### In vitro survival of mycobacteria in the presence of CO and identification of CO resistance gene in Mtb

Although CO toxicity is widespread among diverse bacterial species, Mtb can withstand elevated CO concentrations with only minimal growth inhibition [11]. Under aerobic conditions, when Mtb are treated with CO during log phase, the bacteria are able to effectively resist CO-mediated growth inhibition [11]. Considering that Mtb senses CO in vitro via the DosS/DosT two-component system and its growth in vitro is not severely diminished in the presence of CO (unlike other bacteria when treated with CO), we hypothesized that Mtb CO resistance is genetically encoded. To identify such a gene, we generated an Mtb transposon mutant library and screened for mutants that did not grow in the presence of CO when compared to its growth in the presence of air (Zacharia, et. al, submitted). Interestingly, we identified such a mutant and mapped the transposon insertion to a gene region conserved in mycobacterial species and even phylogenetically distinct organisms such as *Thermatoga maritima* and *Rhodococcus fascians*. To confirm that the newly identified gene does indeed confer CO resistance, Zacharia *et al.* complemented the mutant with the cloned gene of interest, and observed a rescued growth phenotype in the presence of CO (Zacharia, et. al, submitted). Importantly, the mutant’s ability to survive inside wild type macrophages was considerably less than that of wild type Mtb. Moreover, the mutant Mtb strain is attenuated for virulence in a mouse aerosol model of Mtb infection. Thus, host-derived CO can limit Mtb growth in macrophages and mice (Zacharia, et. al, submitted). This discovery of a novel protein involved in CO resistance marks the initial identification of a CO resistance gene in a pathogen. Multiple lines of experimentation are being actively pursued (biochemical, genetic, bioinformatics) to characterize the molecular function of this mycobacterial CO resistance protein to ultimately determine its role in contributing to Mtb pathogenesis.

### Concluding remarks

The effects of CO on bacterial and mammalian cells are diverse including acting as a signaling molecule involved in regulating gene expression [52,53] to serving as a potent, toxic gas capable of inhibiting bacterial growth (Zacharia, et. al, submitted). Amongst human pathogens, *Mycobacterium tuberculosis* is currently the only one known to change its gene expression in response to varying CO concentrations. Some mycobacteria can use CO as a source of energy, but whether Mtb does so during infection remains unknown. However, when host macrophages produce CO Mtb responds by expressing its own CO resistance genes. The ability of Mtb to survive in the presence of CO, in contrast to other known pathogens, indicates that Mtb has uniquely evolved mechanisms to bypass CO toxicity. The identification and characterization of a CO resistance gene and its associated pathways will provide a more comprehensive understanding of Mtb pathogenesis and on a broader scale, host-pathogen interactions.

## Abbreviations

ATP: Adenosine triphosphate; CO: Carbon monoxide; CODH: Carbon monoxide dehydrogenase; CORM: Carbon monoxide releasing molecule; DNA: Deoxyribonucleic acid; dNTP: Deoxyribonucleotide; H_2_O_2_: Hydrogen peroxide; HO: Heme oxygenase; IFN-β: Interferon beta; IRF3: Interferon regulatory factor 3; Mtb: Mycobacterium tuberculosis; NO: Nitric oxide; TNF: Tumor necrosis factor.

## Competing interests

The authors declare no competing interests.

## Authors’ contributions

Both VMZ and MUS conceived of and drafted the manuscript. Both authors read and approved the final manuscript.
